# Pembrolizumab in a Patient With Heavily Pre-Treated Squamous Cell Thymic Carcinoma and Cardiac Impairment: A Case Report and Literature Review

**DOI:** 10.3389/fonc.2020.01478

**Published:** 2020-08-18

**Authors:** Alessandro Cafaro, Alberto Bongiovanni, Valentina Di Iorio, Devil Oboldi, Carla Masini, Toni Ibrahim

**Affiliations:** ^1^Oncological Pharmacy Unit, Istituto Scientifico Romagnolo per lo Studio e la Cura dei Tumori (IRST) IRCCS, Meldola, Italy; ^2^Osteoncology and Rare Tumors Center, Istituto Scientifico Romagnolo per lo Studio e la Cura dei Tumori (IRST) IRCCS, Meldola, Italy; ^3^Radiology Unit, Istituto Scientifico Romagnolo per lo Studio e la Cura dei Tumori (IRST) IRCCS, Meldola, Italy

**Keywords:** pembrolizumab, immune checkpoint inhibitors, thymic cancer, squamous cell, PD-1, PD-L1, cardiotoxicity, case report

## Abstract

Immunotherapy directed at the programmed cell death-1 receptor (PD-1) or its ligand PD-L1 has proven effective in solid malignancies such as melanoma, non-small cell lung cancer (NSCLC), urothelial cancer, renal cell carcinoma, and head and neck cancer. Compared with cytotoxic chemotherapy, radiotherapy, or molecular targeted agents, immunotherapy is an innovative strategy for treating malignancies, with durable clinical responses and manageable adverse events. Thymic carcinomas are extremely rare, constituting only 0.06% of all malignancies, are much more aggressive tumours than thymomas and have a worse prognosis. Nowadays, first line platinum-based chemotherapy for metastatic tumours are the cornerstone of treatment. However, the results of further therapeutic lines for metastatic or relapsed thymic carcinoma are unsatisfactory, with no regimen showing a consistent benefit. Moreover, the rarity of these tumours makes it difficult to carry out clinical trials. Herein we report a remarkable result of 1 year stable disease with good quality of life and no side effects obtained from the use of immunotherapy with pembrolizumab in a case of heavily pre-treated squamous cell thymic carcinoma and cardiac impairment. We also include a literature review of clinical trials on PD-1/PD-L1 inhibitors for the treatment of thymic epithelial cancers, taking a close look at cardiac toxicity.

## Introduction

Based on the RARECARE project definition, thymoma, and thymic carcinomas defined as *Thymic Epithelial Tumours* [TETs] are rare cancers (1). The annual incidence of TETs has been estimated as 1.3–3.2 cases per million worldwide and thymic carcinomas are even rarer, accounting for only 0.06% of all thymic neoplasms (2). Thymic carcinoma (TC) can be histologically classified into several subtypes including squamous, basaloid, mucoepidermoid, sarcomatoid, small cell, adenocarcinoma lymphoepithelial-like, clear cell, and undifferentiated of which the squamous cell subtype is the most common and represents 73–79% of the total cases (3–5). TC is an aggressive tumour with a poorer prognosis than thymoma (6). Five-year survival for locally advanced TC is 36%, decreasing to 24% for metastatic disease (7). Surgical resection of the primary tumour remains the cornerstone of therapy for early-stage disease. In advanced or recurrent TETs, a multimodality approach including surgery, chemotherapy, and radiotherapy is required (8).

Platinum-based chemotherapy remains the gold-standard in the first-line setting. The most frequently used treatment is the platinum-based combination, but fewer than 50% of patients respond (8, 9). Randomized prospective clinical trials are lacking due to the rarity of these tumours, making it important to discuss therapeutic strategies in a multidisciplinary setting. After progression on platinum-based therapy there are limited indications for second-line treatments like sunitinib and everolimus, with data mainly extrapolated from small, single-arm, phase II clinical trials, or retrospective observational studies. VEGF plays a key role in tumour angiogenesis and VEGFR2 and PDGFR-α are known to be activated in thymic carcinoma (10). The activity of sunitinib malate and Lenvatinib, two multi-targeted kinase inhibitors were tested in two phase 2 trials, in which 26 and 38% of patients with thymic carcinoma achieved a partial response, respectively (11, 12). Anthracycline-based chemotherapy is recommended also by the National Comprehensive Cancer Network (NCCN) guidelines but the response rate is similar to that of other regimens and the problem of the best treatment in this setting remains open to debate (11, 13–15). Immuno-oncology (IO) is changing the therapeutic landscape of thoracic malignancies, including TETs, although results are not still not conclusive (16, 17). Second-line pembrolizumab has shown promising activity, Giaccone et al. reporting a median response rate of 22.5% (range 10.8–38.5%) and a median PFS of 4 months (18). However, such findings require confirmation in large prospective clinical trials. Furthermore, PD-L1 expression has been reported in 36–75% of TCs but its prognostic and predictive value remain controversial. Another issue in therapeutic decision-making relating mainly to the choice of IO is the presence of co-morbidities, especially those of a cardiological nature. The incidence of cardiac complications in patients with TC seems to be associated with the use of anthracycline-based chemotherapy and mediastinal radiotherapy (19). Furthermore, myocarditis has been reported in 5–9% of TET patients treated with pembrolizumab (18, 20). Herein we describe the case of a heavily pre-treated patient with squamous cell TC and left ventricular dysfunction in which the administration of the PD-1 antibody pembrolizumab obtained disease control lasting more than a year, without cardiac failure.

## Case Description

**Figure 1:**
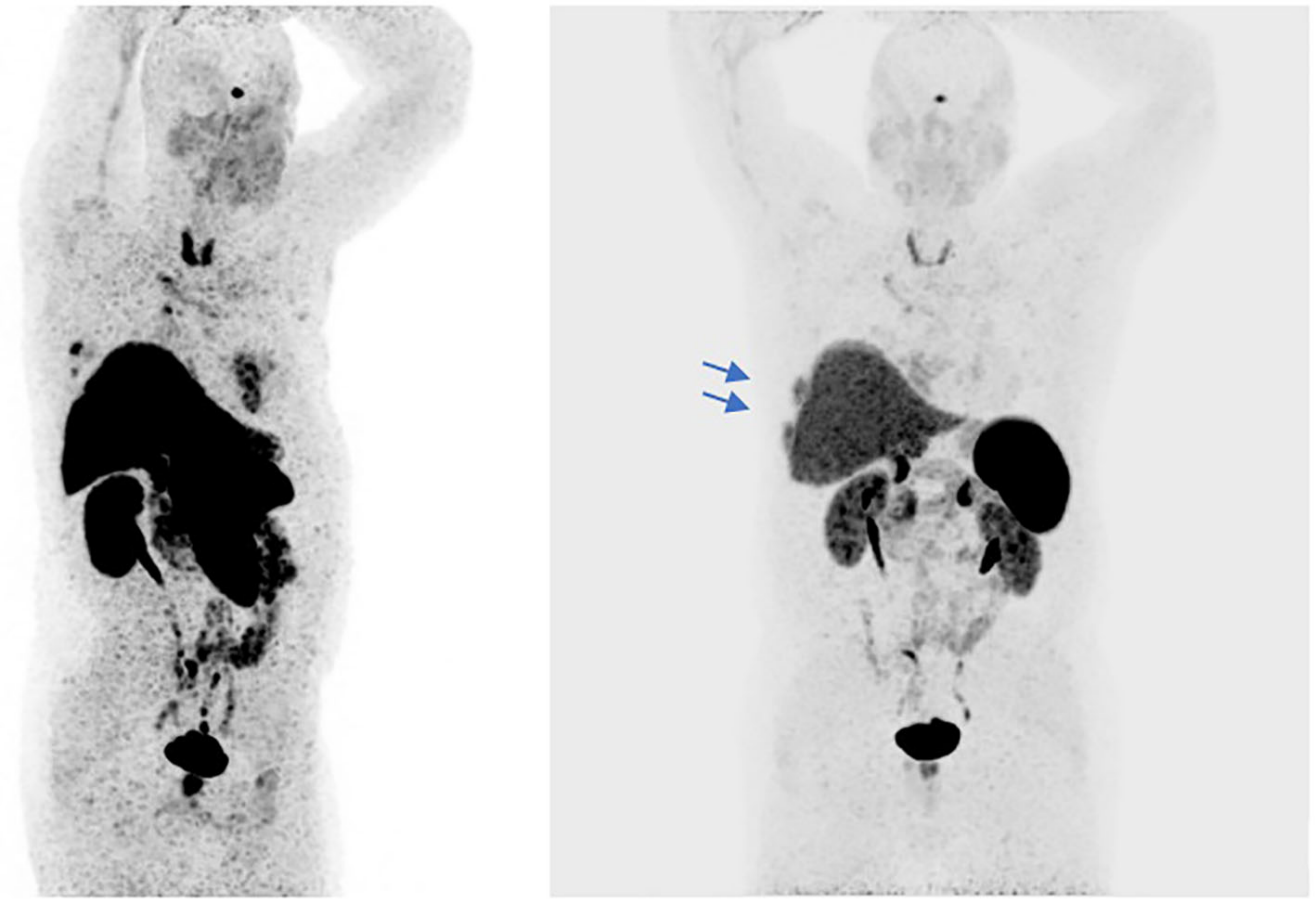
The ^68^Ga-PET/CT shows areas of slight somatostatin receptor uptake in the right subpleural paracardiac area, standardized uptake value (SUVmax = 5.2), and some pre-vascular (SUVmax = 2.3), retrosternal (SUVmax = 3.2), Barety lodge (SUVmax = 3), and right peribronchial (SUVmax = 5.4) node uptake (red arrows). Some areas in the dorsal segment of the upper lobe of the right lung and in middle lobe of the right lung showed no uptake. Slight uptake of the radiopharmaceutical product in the sternal body (SUVmax = 5.9) and the VI and VII right ribs (SUVmax = 8.6) (blue arrows).

**Figure 2:**
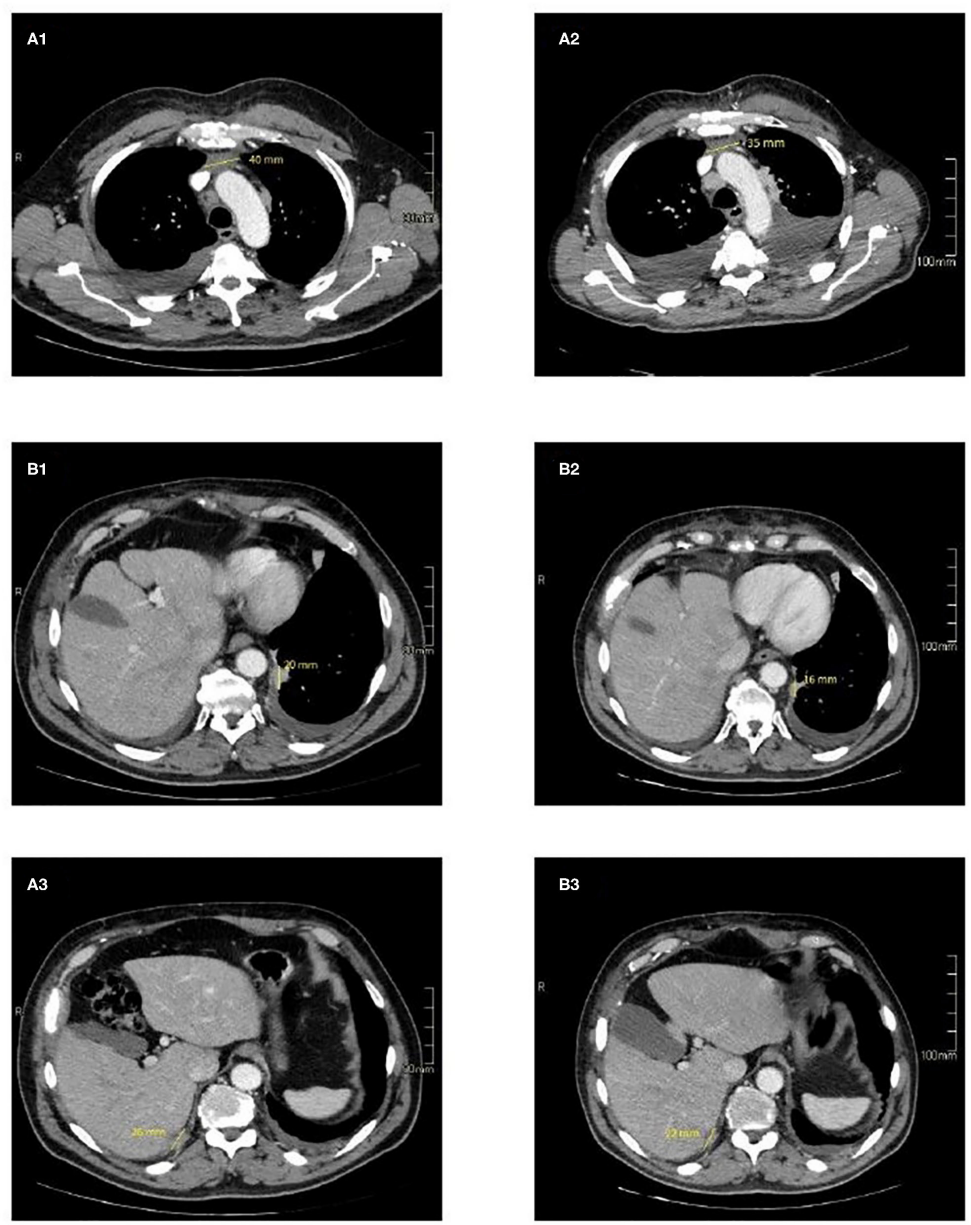
CT imaging of selected target lesions at baseline **(A)** and during treatment **(B)**. Representative axial CT images of patient shows a slight reduction in size of the primitive tumour, 35 mm **(B1)** vs. 40 mm **(A1)** (blue arrows) and pleural localizations on the left side 16 mm **(B2)** vs. 20 mm **(A2)** (yellow arrows), and right side 22 mm **(B3)** vs. 26 mm **(A3)** (red arrows).

In September 2016 a 57-year-old man was diagnosed with a squamous cell TC (diameter 3.6 cm) with involvement of the sternal manubrium and bone metastases (sternum and ribs) (stage IVa according to the Masaoka-Koga system) (21). Eastern Cooperative Oncology Group performance status (ECOG PS) was 0. The patient was a never-smoker with no history of autoimmune disorders. He had mild obesity (BMI: 37 kg/m^2^) as a co-morbidity. From October 2016 to February 2017, six courses of 3-weekly first-line chemotherapy with carboplatin and paclitaxel were administered. After three cycles the patient obtained a partial response (PR) according to RECIST (Response Evaluation Criteria in Solis Tumours) 1.1, confirmed at the end of the sixth cycle (22). Sequential radiotherapy was performed on the mediastinal mass (60 Gy in 30 fractions). Four months after the end of the first-line treatment an ^18^FDG-PET/CT (18-fluorodeoxy-glucose positron emission tomography-computed tomography) scan showed areas of increased uptake in the 6th and 7th right ribs and progressive disease in the sternal body according to MD Anderson criteria (23). From July to December 2017 the patient underwent second-line therapy with gemcitabine, but an ^18^FDG-PET/CT revealed progression of the primary mediastinal mass, new lesions in the posterior arch of the 6th right rib and pulmonary hilar lymph node involvement. A CT scan confirmed these findings. On January 2018 the patient was referred to our institute for a second opinion. After a multidisciplinary discussion, it was decided to perform a 68-Gallium (68Ga)-DOTATOC-PET/CT, which showed very slight somatostatin receptor uptake by both the primary and metastatic lesions (Figure 1). This excluded the patient from being enrolled in a phase II trial for tumours expressing somatostatin receptors type 2 (sstr2) with Luthatera^®^ peptide receptor radionuclide therapy (NCT03454763). Given the previous treatment received and the good ECOG PS, third-line chemotherapy with a modified ADOC (mADOC) scheme (doxorubicin 40 mg/m^2^, vincristine 0.60 mg/m^2^, cyclophosphamide 700 mg/m^2^) without cisplatin was started in February 2018, obtaining a PR after the third cycle. The cardiac evaluation performed before treatment had shown a normal heart function with a left ventricular heart fraction (LVHF) of 61%. In July 2018 there was a reduction in LVHF (49%) with mild symptoms (class 2 according the New York Heart Association Classification) caused by the previous treatment and general conditions, and mADOC treatment was stopped. Echocardiography showed concentric left ventricular hypertrophy, cardiac hypokinesia and a further decrease in LVHF (43%). In November 2018 a CT scan documented further disease progression with the appearance of multiple bilateral pleural and pulmonary lesions and suspected liver metastases. Molecular evaluation of PD-L1 expression and other mutations was requested but not performed because of insufficient bioptic tissue. After a multidisciplinary team discussion with the institute's cardio-oncologist, the patient was deemed unamenable to treatment with sunitinib given the known cardiotoxicity of tyrosine kinase inhibitors (TKIs) and the risks associated with its use in a patient with the aforementioned comorbidity. In February 2019, given the good clinical conditions and the relatively young age of the patient, it was decided to start off-label fourth-line treatment with pembrolizumab (200 mg every 3 weeks) (approved by the Italian Medicines Agency in accordance with Art. 48, Leg. 326/2003) and monitor cardiac side-effects. The beta 1-selective-adrenoceptor blocking agent bisoprolol and an angiotensin-converting enzyme inhibitor (ramipril) were added by the cardio-oncologist. The patient underwent 18 courses of three-weekly pembrolizumab, obtaining stable disease for more than 1 year (Figure 2).

**Figure 3:**
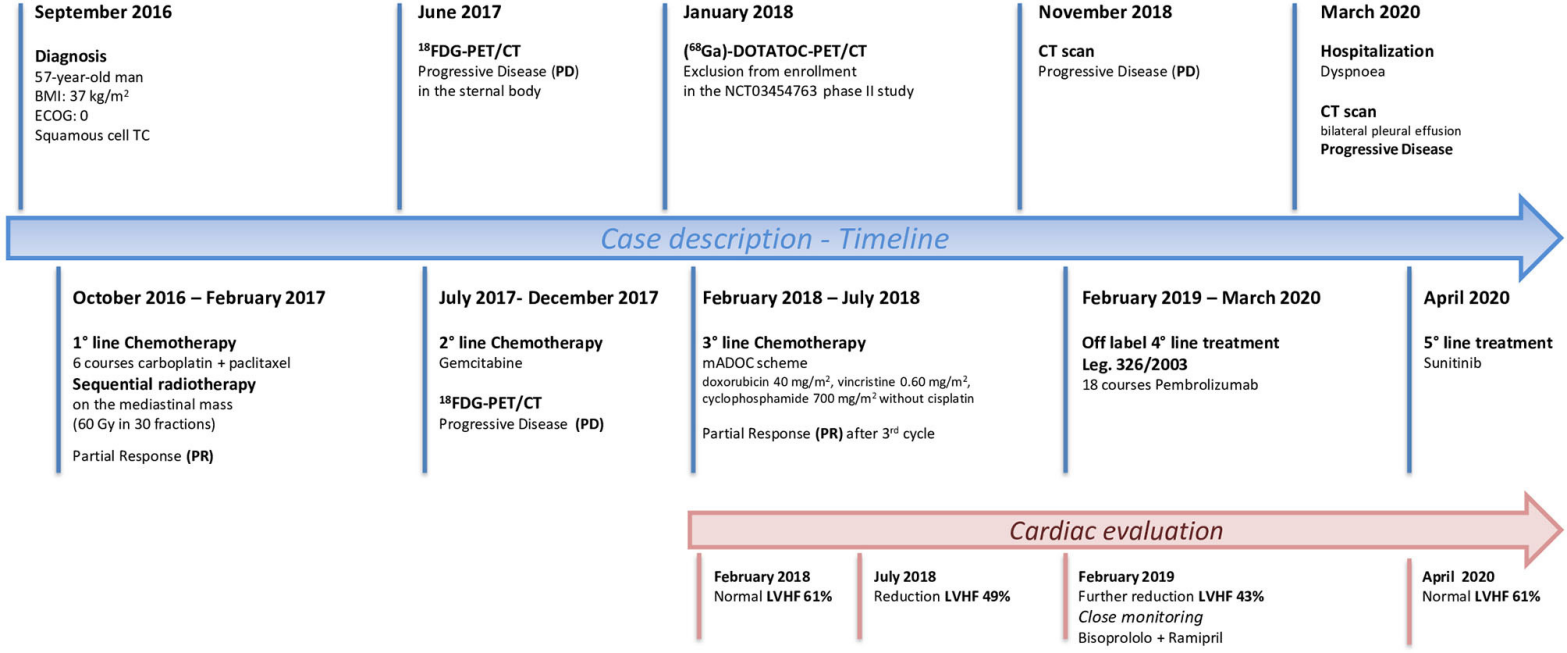
Timeline showing patient history from the diagnosis of Squamous Cell Thymic Cancer to the most recent treatment perspectives.

During this period, cardiac function was monitored closely (echocardiography and blood tests) to detect even the slightest sign of worsening heart failure. Levels of myosin light chain troponin, N-terminal prohormone of brain natriuretic peptide (NT-proBNP), lactate dehydrogenase and creatine phosphokinase were normal and monitored over time. No signs of heart damage were detected and no adverse events were reported. In March 2020 the patient was hospitalized for dyspnoea, a CT scan showing bilateral pleural effusion. More than 2,000 mL of exudative fluid was drained by thoracentesis and cytology was positive for TC cells. Another CT performed in April confirmed disease progression according to immune-related RECIST, with an increase in the size of the primary tumour and pleural localizations. Echocardiography of April 2020 revealed an improvement in cardiac function, with normal LVHF (62%). It was decided to stop pembrolizumab and after a multidisciplinary discussion the patient started treatment with sunitinib (Figure 3).

## Discussion

Several studies have investigated the role of IO in TCs. Although PD-L1 is mainly expressed in cortical and medullary thymic epithelial cells (24), Padda et al. observed that high PD-L1 scores were found more often in TETs than in normal thymus tissue and that its expression was inversely related with clinical outcome (25). On the basis of these findings, the blockage of the PD-1/PD-L1 axis by specific monoclonal antibodies (MAB) could be a promising treatment for TC.

The efficacy of the anti PD-L1 pembrolizumab was evaluated by Giaccone et al. in 40 patients with recurrent TC in progression after at least one line of chemotherapy in a single-arm, single centre, phase II study. Patients had received a median of two previous therapies. Nine (23%) patients achieved an objective response, one a complete response (CR) and 8 PR and 21 (53%) stable disease (SD). Medium progression-free survival (PFS) was 4.2 months (95% CI 2.9–10.3) and was longer in patients with high PD-L1 expression [TPS (tumour proportion score) ≥50%] than in those with low or no PD-L1 expression (median PFS of 24 months, 95% CI 5.8–42.3 vs. 2.9 months, 95% CI 1.7–4.1). The disease control rate (DCR) was 75%, higher than that seen in Sunitinib study and 3 of 21 patients who obtained a SD maintained this response for more than a year (11, 19). A head to head comparison should clarify these findings.

Another phase II trial tested pembrolizumab in 33 patients with TET in whom platinum-based systemic chemotherapy was unsuccessful. The overall response rate was 21%, disease control rate (DCR) 79%, and median PFS was 6.1 months. High PD-L1 levels were confirmed as a significant predictive marker of response to therapy (21). Conversely, Katsuya et al. reported conflicting results in a phase II trial evaluating nivolumab in 15 patients with previously treated TC, six of whom had received more than 3 previous systemic treatments. Notably, no data about PD-L1 TPS were reported. No objective responses were observed and patient recruitment was stopped after a pre-planned futility interim analysis. The DCR was 73% and the median PFS 3.8 mouths (26). Finally, data on the safety and activity of the anti PD-1 avelumab have recently been reported in patients with advanced thymoma treated with at least one prior standard therapy. Of the 7 patients with B1–B3 thymoma, 2 had confirmed PR, 2 unconfirmed PR, 2 SD, and one disease progression (27).

Results from different clinical trials have shown the promising activity of Immune checkpoint inhibitors (ICI) targeting the PD-1/PD-L1 axis in patients with advanced TC (Table 1) and NCCN guidelines recently added pembrolizumab as a potentially effective drug for second-line therapy in TC. Considerable activity has been noticed in subgroup of patients with high tumour PD-L1 levels.

**Table 1:** Immunotherapies in thymic tumours.

**References**	**Patients**	**T; TC (SCTC)**	**Drug**	**ORR %**	**DCR %**	**mPFS**	**mOS**	**Cardiac side-effect**	**Grade**
Giaccone et al. (18)	40	0; 40 (19)	Pembrolizumab	22.5	75.5	4.2 mo (8.9–10.3)	24.9 mo (15.5–NR)	5% (2 patients–myocarditis)	4
Cho et al. (20)	33	7; 26 (NRep)	Pembrolizumab	T: 28.6 TC: 19.2	T: 100 TC: 73	6.1 mo	–	9.1% (3 patients–myocarditis)	≥3
Katsuya et al. (26)	15	0; 15 (13)	Nivolumab	0	73	3.8 mo (1.9–5.6)	NA (11.3–NA)	0 %	–
Rajan et al. (27)	8	7; 1 (NRep)	Avelumab	T: 29 TC: 0	T: 87 TC: 100	–	–	37.5% (3 patients–elevated troponin)	1

*DCR, disease control rate; mo, months; mPFS, median progression-free survival; mOS, median overall survival; NR, not reached; ORR, objective response rate; pts, patients; T, thymoma; TC, thymic carcinoma; NRep, not reported; SCT, squamous cell thymic carcinoma; NA, not available*.

Immune-related toxicity can affect potentially any organ, in particular the cardiovascular system (28). Myocarditis is a serious autoimmune condition. ICI-related myocarditis typically develops during the early stages of treatment (17–34 days after initiation of ICI therapy) and can show a rapid course, with severely reduced LV function, hemodynamic instability, and the need for intensive care (29). Although rarely described in the literature it has been reported in 5% of patients with TC and in 43–57% of patients with thymoma recruited in clinical trials and treated with ICIs (Table 1). Little is known about the underlying pathogenetic mechanisms of immune-related toxicity. Recent data suggests that the tumour itself may play an important role in mediating an immune response against cardiac structures due to epitopes shared between the myocardium and the tumour. In TETs, the incidence of myocarditis is higher in thymomas characterized by autoimmune disorders than in TCs (30).

In the case of our patient, literature data, tumour histology (squamous cell TC vs. thymoma), the previous therapeutic lines administered and the absence of autoimmune disorders prompted us to proceed with pembrolizumab, despite the unknown expression level of PD-L1 in tumour cells. Given the heavy pre-treatment and comorbidity of our patient, we obtained an excellent result. The PFS of more than 1 year is between 2- and 3-fold longer than the median PFS reported in the aforementioned clinical trials on anti PD-1/PD-L1 antibodies. In addition, our patient did not experience any cardiac adverse effects. The results obtained also stress the importance of a multidisciplinary team approach, thanks to which a safer and more suitable therapeutic strategy was chosen for our patient. The treatment not only led to a good clinical result but has also enabled us to proceed with an additional therapeutic line (sunitinib) thanks to the recovery of normal cardiac function following the damage caused by previous chemo- and radio-therapy.

## Conclusions

We presented the case of a patient with squamous cell TC and multiple metastatic sites, unsuccessfully treated with three prior systemic therapy, in whom we obtained good disease control from the off label use of the anti PD-1 antibody pembrolizumab. Our findings and literature review suggest that anti PD1/PD-L1 drugs could be a novel treatment for TC with promising activity and safety, despite the few data available from small monocentre trials. Further research on phase III multicentre clinical trials is warranted to investigate the efficacy and safety anti PD-1/PD-L1 drugs.

## Ethics Statement

The studies involving human participants were reviewed and approved by Comitato Etico della Romagna - CEROM. The patients/participants provided their written informed consent to participate in this study. The authors obtained patient consent for publication of clinical data and images.

## Author Contributions

AC and AB drafted the manuscript and carried out the literature review. DO provided the images and the pathology review. TI supervised the study. CM and VD revised the manuscript for intellectual content. All authors read and approved the final version of the manuscript for submission.

## Conflict of Interest

The authors declare that the research was conducted in the absence of any commercial or financial relationships that could be construed as a potential conflict of interest.
